# **Migration, Retention and Return Migration of Health Professionals** Comment on "Doctor Retention: A Cross-sectional Study of How Ireland Has Been Losing the Battle"

**DOI:** 10.34172/ijhpm.2020.225

**Published:** 2020-11-23

**Authors:** Latha S. Davda, David R. Radford, Jennifer E. Gallagher

**Affiliations:** ^1^University of Portsmouth Dental Academy, Portsmouth, UK.; ^2^Faculty of Dentistry, Oral and Craniofacial Sciences, King’s College London, London, UK.

**Keywords:** Retention, Migration Motivation, International Recruitment

## Abstract

Medical education and training of health professionals are linked with their recruitment and retention. Practising as a competent health professional requires life-long continuous training and therefore training structures in health systems appear to influence doctors job satisfaction, their well-being and their intentions to remain in that health system. The commentary critiques aspects of the paper on doctors retention in Ireland, while drawing some parallels with the United Kingdom. There appears to be an emerging type of health professional migrants ‘education tourists’ who travel to other countries to obtain medical education creating new routes of migration and this presents new challenges to source and destination countries. The global shortage of doctors and other health professionals further exacerbates health inequalities as seen in the present pandemic and therefore the increased need for research into health professionals’ migration and their integration.

## Background

The retention of human resources for health in the country where they train and qualify is an important and complex issue that is influenced by global, national, regional policies and individuals’ freedom and professional choices.^1^ Globalisation with ease of movement, global recognition of professional qualifications, internationalisation of medical and dental education and a net global migration of people, goods and knowledge has led to new challenges for governments. Public investment in training the health professionals and the cost benefit of these investments is important.^2^ Retention is a concern for low and middle-income countries already facing shortage of health professionals, however, this paper by Brugha et al^3^ highlights retention as a concern for The Republic of Ireland, a high income destination country for migrant health professionals. It is also recognised that some countries such as Ireland, Gulf countries and the United Kingdom act as stepping stones for further migration thereby increasing the complexity of accurately forecasting the workforce retention in their health sector. The traditional migration of health professionals from low and middle income countries to high income countries in search of better incomes, livelihood and training may result in onward migration to final destination countries mainly the United States, Canada and Australia.^4^ High income countries have resorted to active recruitment of health professionals from other countries rather than invest and increase the number of domestically trained professionals.^2,5^ Active recruitment and better funded National health systems whose training is sought after, are natural pathways that trigger and facilitate migration from low and middle income countries. Neo-liberalisation of medical education in several countries attracts international students who expect to stay in that country to work at least for a short period of time after graduation. Certainly the concept of being a global citizen was strong among dentists who have migrated to the United Kingdom.^6^

 The Republic of Ireland was highlighted as one of the countries where there is a high flux of healthcare professionals and therefore an important study country to investigate the impact of policy levers used to shape the health labour market. The doctors retention strategy was a five year programme (2015 to 2020) and it appears to have failed in its aim to retain overseas as well as home educated doctors.

## Summary of the Study

This cross-sectional study (Brugha et al)^3^ thus aimed to identify the characteristics and patterns of doctors who were non-consultant hospital doctor trainees who planned to emigrate further, to inform measures to better retain these doctors. Data were collected through an online survey from November 2017 to February 2018. The structured questionnaire included sections on professional characteristics, demographics, training and working experiences rated on a Likert scale, with an additional question on their future career intentions of 4 possible intentions “remain in Ireland,” “go abroad but return,” “go abroad and not return” or “leave medicine.”

 The response rate was 28% as 1468 doctors responded. However, the response rate dropped to 22% (total 1148) as 320 participants did not complete the question on their future career intentions. 45% of the respondents planned to ‘stay in Ireland,’ 35% to ‘go abroad but return,’ 17% to ‘migrate and not return’ and 3% to ‘leave medicine.’ Females reported that they were more likely to remain (48% vs. 41%) and single respondents were more likely than those who were married/cohabiting to have a period of time abroad (43% vs. 29%), as were those without children. The authors state that from their findings, the typical doctor who reported an intention to migrate for a period of time was, under 30 years old, male, single with no dependents (the backpacker migrant). This is contrasted with those who wished to migrate and not return as over 30 male, non-Irish, married/cohabiting, had dependent children and was a graduate entry student.

## Commentary

The study is a well considered piece of work from a country that despite having implemented a national doctor retention scheme since 2014 has not tackled the fundamental cause of outward migration of either its own medical graduates or international doctors, many from poorer countries. This has been attributed to low staffing levels, stressful working conditions that go ‘hand in hand’ and poor training experiences. This is not a unique situation and has also been recognised by the UK General Medical Council which stated that approximately 25% to 50% of UK doctors reported longer working hours, poor work-life balance, lack of support and poor mentoring.^7^ Ireland can be considered as a microcosm, subject to global developments due to a global estimated shortfall by 2030 of 750000 doctors in 31 Organisation for Economic Co-operation and Development (OECD) countries, which incredibly excludes an estimated shortfall of 2600000 doctors in less economically developed countries, currently being highlighted by the coronavirus disease 2019 (COVID-19) pandemic.^8^

A further element of this outward migration is due to the doctors viewing themselves as global citizens with a highly portable medical qualification especially to anglophile countries. The ‘back packer’ migrant (young and single) and dissatisfied graduate entry doctors (more mature) were also likely to migrate and not return. A desire and access to international education and hence ‘education tourist’ type of migration was seen in some dentists migrating to the United Kingdom^6^ and more research is needed in this new type of migrant who have invested time and money into their medical education with the hope that this will act as a facilitator for migrating to a high income country.

 The response rate of 22% of the total data base was considered reasonable by the authors citing the difficulty of achieving high response rates among doctors. However, this response rate of just one fifth of those in training has to be taken into account when interpreting their career intentions. The responders may be the ones who were disadvantaged due to lack of career progression in the health system and ‘potentially discriminated’ as they reported bullying and harassment.

The complexity of the findings under-line the complexity of respondents push and pull factors that influence their reported intentions to remain, migrate either for a certain period or permanently. This was reflected in the key messages of the paper in a call for a more diversified retention strategy that could facilitate those who undertake speciality training abroad but could return to permanent (senior) position in Ireland. It was not surprising that more than quarter (n = 154, 27%) elected to go the United Kingdom. Ireland and the United Kingdom are bound by a special agreement under the Common Travel Area, allowing health workforce movement, irrespective of its relationship to the European Economic Area since 1921, a relationship that was reaffirmed in 2019.^9^

Intention to migrate studies are not very reliable indicators of actual migration, however they are a good indicator of dissatisfaction and aspirations of health professionals.^10,11^ Difficulties in recruitment and retention of dentists in some rural, deprived or inner city areas has resulted in closure of dental practices in certain areas of the United Kingdom, post-Brexit indicating this is a challenge for all health sectors.^12^

Practices of undertaking short international placement or electives may be one of the ways of encouraging short mobility which will substantially support the doctors progression to consultant or higher academic positions, and enhance their working practices.^13^ Global exposure could improve the culture referred to in the paper, in turn may make it more attractive to work in Ireland. Those who graduate as doctors are considered to belong to the social elite and usually have more money and position in the society and naturally this could give them the ability to exercise free will and to move from small towns to cities or migrate outside the country. In a small island such as Ireland, the doctors workforce is a comparatively small elite group with close networks which could potentially make it difficult for those without networks to progress, which is another reason why they may wish to migrate further. It may be that policy-makers must accept that there will be outward mobility among doctors and therefore strive to make working in Ireland more attractive, thus desirable to remain and more welcoming to those who return.

Increased demand for doctors worldwide is being driven by demographic changes with aging populations and increased demand for more complex care along with increases in urban populations and rising expectation in medium income countries. In turn this places increased workload on doctors leading to possible burnout and mental health issues. The COVID-19 pandemic has forced all countries to look closely at their health systems. The paper ends with a comment that graduates have returned home to offer their services during pandemic. This may be true for some doctors: however, severe restriction to travel has forced several health professionals to remain stranded in destination countries. Migrant doctors and other health professionals who are employed on temporary contracts, working in least desired posts are likely to be the first causalities in this pandemic, with regards to their jobs and risks to their health.^14^

The decision to return may be as complex as the decision to migrate and appears to be more dependent on the socio-cultural integration in the destination country than economic integration, however the pandemic may change this. Transnational migrants, who maintain a bond to their country of origin in the form of investments, frequent travel, professional connections make it more likely for individuals to return.^15^

## Ethical issues

 Not applicable.

## Competing interests

 Authors declare that they have no competing interests.

## Authors’ contributions

 LSD designed, analysed the paper and wrote the first draft. DRR involved in critical analyses of the paper and revising drafts and references. JEG contributed to the intellectual critiquing in the commentary and approval of the final draft.

## Authors’ affiliations

^1^University of Portsmouth Dental Academy, Portsmouth, UK. ^2^Faculty of Dentistry, Oral and Craniofacial Sciences, King’s College London, London, UK.
